# Divergent Occurrence of Carotid Intima-Media Thickness and Carotid Arteries Plaques in Stable Kidney Transplant Recipients

**DOI:** 10.31083/j.rcm2312386

**Published:** 2022-11-28

**Authors:** Aureliusz Kolonko, Rafał Ficek, Beata Styrc, Michał Sobolewski, Roksana Stankowska, Jerzy Chudek, Andrzej Więcek

**Affiliations:** ^1^Department of Nephrology, Transplantation and Internal Medicine, Medical University of Silesia in Katowice, 40-027 Katowice, Poland; ^2^Department of Internal Medicine and Oncological Chemotherapy, Medical University of Silesia in Katowice, 40-027 Katowice, Poland

**Keywords:** atherosclerosis, biomarkers, calcified plaques, kidney transplantation, ultrasound

## Abstract

**Background:**

Carotid atherosclerosis is one of the main cerebrovascular complications in kidney transplant recipients (KTRs). We analyzed the relationships between carotid intima-media thickness (IMT) and the occurrence and characteristics of carotid plaques in a cohort of KTRs.

**Methods:**

In 500 KTRs (aged 49.9 ± 12.0 years), IMT was measured and carotid plaques were semi-qualitatively assessed. Concomitantly, biochemical and hormonal inflammatory, vascular and calcium-phosphate metabolism parameters were also assessed.

**Results:**

In 10.2% of patients, a side-to-side IMT difference >0.1 mm was observed, whereas 26.8% of patients with no plaques in one carotid artery had at least one contralateral calcified plaque. Multivariate logistic regression analysis revealed that age ($r_{\text{partial}}$ = 0.409; *p *< 0.001), male sex ($r_{\text{partial}}$ = 0.199; *p *< 0.001), and coronary artery disease ($r_{\text{partial}}$ = 0.139; *p *< 0.01) independently increased IMT ($R^{2}$ = 0.25). For the occurrence of calcified carotid plaques, age ($r_{\text{partial}}$ = 0.544; *p *< 0.001), male gender ($r_{\text{partial}}$ = 0.127; *p *< 0.05), and the duration of renal insufficiency prior to transplantation ($r_{\text{partial}}$ = 0.235; *p *< 0.001) were confirmed as independent variables.

**Conclusions:**

Substantial side-to-side differences in IMT values and carotid plaques distribution are present in a large percentage of stable KTRs. In addition, there are different clinical risk factors profiles associated with IMT and the presence of calcified plaques. Vascular and calcium-phosphate metabolism biomarkers were not associated with any carotid atherosclerosis characteristics.

## 1. Introduction

Carotid atherosclerosis is one of the major cardiovascular (CV) risk factors for the occurrence of an ischemic stroke [1, 2]. Traditional risk factors associated with atherosclerosis are age, male gender, smoking, dyslipidemia, hypertension, and diabetes mellitus [3]. In addition, chronic kidney disease (CKD) has also been shown to be associated with greater carotid intima-media thickness (IMT) and the occurrence of symptomatic ischemic stroke [4, 5] as well as with increased carotid artery stiffness and the presence of calcified plaques [6]. As a consequence, CV mortality is the major cause of death in CKD patients [7]. After successful kidney transplantation, despite the reduction of some risk factors (left ventricular hypertrophy, hypertension), CV disease remains a leading cause of death despite a functioning graft [8].

Quantitative evaluation of atherosclerosis with B-mode ultrasound involves the measurement of carotid intima-media thickness (IMT) and the assessment of carotid plaques. Both these measurements are biologically distinct entities and represent different phenotypes of atherosclerosis [9]. IMT is mainly reflective of hypertensive medial hypertrophy and is only weakly associated with traditional coronary risk factors whereas plaques are more strongly associated with traditional risk factors [10]. In patients with end-stage renal disease, the adverse CV consequences of hyperphosphatemia are most likely mediated via its ability to enhance the development of vascular calcifications [11]. CKD also significantly affects plaque composition [12]. Moreover, in CKD patients the rate of atherosclerotic plaque formation is a strong, independent predictor of CV events [13].

In clinical practice, we observed a significant asymmetry of carotid plaques and a substantial discrepancy between IMT values and the plaque burden in some stable kidney transplant recipients (KTRs). In our previous investigations, several clinical measures and biochemical markers were assessed in three different KTRs cohorts [14, 15, 16]. Based on our prospective kidney transplant database, we retrospectively analyzed IMT and the presence of plaques as markers of carotid atherosclerosis, as well as numerous biochemical and hormonal parameters.

## 2. Materials and Methods

### 2.1 Study Participants

This study enrolled 500 KTRs who attended our out-patient clinic from 2013 to 2017, in whom carotid artery ultrasound with IMT measurement and carotid plaque assessment were performed. Those examinations were part of the protocols of our previous clinical studies [14, 15, 16], and were approved by the Bioethics Committee of the Medical University of Silesia. All participants gave their written informed consent. The study was conducted in accordance with the Declaration of Helsinki. In addition to data retrieved from the prospective transplant center patient registry, carotid ultrasound, including the assessment of IMT and carotid plaques were performed.

Patients were identified as active smokers, when they were currently smoking or they declared the period of non-smoking as being shorter than 5 years.

### 2.2 Clinical and Anthropometric Measurements

Body weight and height were measured following standard procedures, and BMI was calculated in kg/$m^{2}$.

Office arterial blood pressure (OBP) was measured three times in the sitting position in the arm without vascular access during the physical examination. Patients whose OBP was equal or above 140/90 mmHg or those who received antihypertensive medication were diagnosed as hypertensives.

Diabetes was diagnosed in accordance with the American Diabetes Association criteria [17].

The duration of renal function insufficiency was estimated, based on the data collected at the time of kidney transplantation (the period of time since the first elevated serum creatinine concentration to the kidney transplantation procedure).

### 2.3 Laboratory Measurements

Routine laboratory measurements were performed in the hospital laboratory (Synchron Cx-9, Beckmann Coulter Inc., Fullerton, CA, US). Plasma high-sensitivity C-reactive protein (CRP) concentration was measured by nephelometry (Siemens Healthcare Diagnostics, Deerfield, IL, US) with a limit of quantification (LoQ) of 0.02 mg/L. Intact plasma parathormon (iPTH) concentration was measured using the immunoassay method (Abbott Diagnostics, Abbott Park, IL, US) with a LoQ <3 pg/mL, intra-assay variation <6/1% and inter-assay variation <6.4%, whereas plasma concentrations of interleukin 6 (IL-6) and tumor necrosis factor alpha (TNF-α) were measured by ELISA (R&D System, Minnesota, MN, US) with a LoQ 0.7 pg/mL and 6.23 pg/mL, intra-assay variation <4.2% and 3.0%, and inter-assay variation <6.4% and 8.4%, respectively. Plasma concentrations of osteoprotegerin (OPG) were measured with the use of an immunoassay (Microvue Bone Health; Biovendor Laboratory Medicine, Modrice, Czech Republic) with a LoQ 0.03 pmol/L, intra-assay variation <3.5% and inter-assay variation <5.8%. Plasma concentrations of asymmetric dimethylarginine (ADMA) and oxidized LDL (ox-LDL) were measured using ELISA (Immundiagnostik, AG, Bensheim, Germany), with a LoQ of 0.16 μmol/L and 0.0205 U/mL, intra-assay variation <7.6% and <6.9% and inter-assay variation <4% and <14.4%, respectively. Plasma concentrations of endothelin-1 (ET-1) were measured using ELISA (USCN Life Sciences, Wuhan, People’s Republic of China), with a LoQ of 2.71 pg/mL, intra-assay variation <10%, inter-assay variation <12%.

### 2.4 Carotid Sonography

Carotid ultrasound was performed using a Siemens machine (Sonoline Antares, Mountain View, CA, USA), equipped with a 4.0–9.0 MHz linear transducer. Carotid arteries were examined with the patient in the supine position with the neck extended. The evaluation included the common, internal, and external carotid arteries, and the carotid bifurcation on each side. The common carotid artery intima-media thickness (IMT) was measured manually within 2 cm proximal to the carotid bulb, omitting any visible plaques. At longitudinal scans, the distal lines representing lamina intima and media were sharply visualized and the electronic calipers were placed to perform the exact IMT measurement. The accuracy of the single measurement was 0.5 mm and 3 consecutive measurements were made on each side, then the results were averaged. The highest value on both sides values was reported as the maximal IMT value. At each location, the carotid bulb and preceding common carotid artery were carefully evaluated in terms of the presence of plaques, which was classified based on the simplified scale: 0—no lesions, 1—non-calcified lesions, 2—at least one calcified lesion, 3—few calcified lesions, 4—carotid bulb heavily covered by calcified lesions. A final plaque score was equal to the highest score from both sides. All carotid sonographic examinations were performed by single investigator (AK).

### 2.5 Data and Statistical Analysis

Post-transplant major adverse cardio- or cerebrovascular events (MACE) were defined as the incidence of myocardial infarct, stroke, or cardiac artery stenting/surgical revascularization.

Kidney graft function was measured by the estimated glomerular filtration rate (eGFR) calculated according to the Modification of Diet in Renal Disease (MDRD) formula.

Statistical analyses were performed using the STATISTICA 13.3 PL for Windows software package (Tibco Inc., Palo Alto, CA, USA) and MedCalc 18.6 (MedCalc Software, Ostend, Belgium). Values are presented as means and 95% confidence intervals or medians with Q1–Q3 values, as appropriate, or frequencies. Comparisons were performed between 2 groups based on the mean value of maximal carotid IMT and between 3 groups, defined by the presence and type of carotid plaques. Based on the presence and type of carotid artery plaques, all study participants were assigned to the subgroup 1 (no plaques), subgroup 2 (only non-calcified plaque/plaques) or subgroup 3 (one or more calcified plaque). For these comparisons, the Student *t* test and the analysis of variance test (for quantitative variables) or the χ^2^ test (for qualitative variables) were used accordingly. For variables with non-parametric distribution, the Mann-Whitney U test or the Kruskal-Wallis test was used. Receiver operator characteristics (ROC) analysis was applied to determine the cut-off values for age and the duration of renal insufficiency, associated with the presence of calcified carotid lesions. Calculation of correlations were done using the Spearman coefficient.

Multivariate backward regression analysis was performed for the variability of IMT value, including potential explanatory variables: age, sex, the presence of coronary artery disease, smoking status, hemoglobin level and the presence of calcified carotid plaques. Multivariate models included variables selected on the basis of group comparison and univariate logistic regression analyses. The stepwise selection method was used.

Multivariate backward regression analysis was also performed for the presence of calcified plaques as dependent variable, including potential explanatory variables: age, sex, BMI, the presence of hypertension, pulse pressure, IMT, coronary artery disease or MACE, smoking status, serum glucose or the presence of hyperuricemia. Another multivariate backward regression analysis was performed for the presence of calcified lesions in a subset of 319 and 146 patients, respectively, i.e., in a cohort of patients with available results of relevant biochemical markers, and included age, the number of antihypertensive drugs, CRP, OPG and sclerostin levels as potential independent variables. In all the statistical tests, the ‘*p*’ values below 0.05 were considered statistically significant.

## 3. Results

### 3.1 Study Group

The study group consist of 500 stable KTRs, whose clinical characteristics are presented in Table 1. Mean age at the time of the study was 49.9 ± 12.0 years. Median time after kidney transplantation was 86 (Q1–Q3, 65–117) months. The causes of end-stage renal disease were: glomerulonephritis (48%), diabetes mellitus (13.6%), pyelonephritis (11.6%), autosomal dominant polycystic kidney disease (8.4%), hypertensive nephropathy (5.6%), other and unknown (12.8%). 97.4% of patients received their organ from a deceased donor. Most of the patients received immunosuppression therapy with cyclosporine A or tacrolimus, anti-metabolic drugs (mainly mycophenolate mofetil or mycophenolate acid), and steroids.

**Table 1.** **Clinical characteristics of study group**.

Parameter		Value
		N = 500
Age at the time of the study [years]		49.9 (48.8–51.0)
Gender [M/F]		284/216
BMI [kg/$m^{2}$]		26.3 (25.9–26.7)
Dialysis vintage [months]*		25.0 (14.0–42.0)
Time after transplantation [months]*		86.0 (65.0–117.0)
Retransplant [n (%)]		42 (8.4)
Hypertension [n (%)]		445 (89)
MAP [mmHg]		100.5 (99.5–101.4)
Pulse pressure [mmHg]*		50.0 (40–60)
Number of antihypertensive drugs [n]*		2 (1–3)
Structure of hypertensive treatment [n (%)]		
	ACE-I/ARB	143 (28.6)
	Beta-blocker	348 (69.6)
	Ca-blocker	207 (41.4)
	Diuretics	142 (28.4)
Diabetes [n (%)]		137 (27.4)
Coronary artery disease [n (%)]		63 (12.6)
Previous MACE [n (%)]		54 (10.8)
Smoking status [%]		89 (17.8)
eGFR [mL/min/1.73 $m^{2}$]*		49.4 (36.4–68.0)
Proteinuria ≥1 g/24 h [n (%)]		35 (7.0)
Glucose [mmol/L]*		5.0 (4.7–5.7)
Calcium [mmol/L]*		2.4 (2.3–2.5)
Phosphate [mmol/L]*		1.0 (0.9–1.2)
iPTH [pg/mL]*		107 (69–174)
Cholesterol [mmol/L]		5.3 (5.2–5.4)
Triglycerides [mmol/L]*		1.5 (1.0–2.1)
Hyperlipidemia [n (%)]		265 (53)
Hyperuricemia [n (%)]		286 (57.1)
Hemoglobin [g%]		13.5 (13.3–13.7)
Main medications [n (%)]		
	Statins/fibrates	135 (27)
	Calcineurin inhibitors [CyA/Tc]	238 (48)/246 (49)
	Glucocorticoids	305 (61)
	Calcium carbonate	74 (14.8)
	Vitamin D	94 (18.8)

Data presented as means and 95% Confidence Intervals or frequencies, except * medians and Q1–Q3 values. BMI, body mass index; MAP, mean arterial pressure; ACE-I, angiotensin converting enzyme inhibitor; ARB, angiotensin receptor blocker; MACE, major adverse cardio- and cerebrovascular event; eGFR, estimated glomerular filtration rate; iPTH, intact parathormon; CyA, cyclosporine A; Tc, tacrolimus.

### 3.2 Intima-Media Thickness

In the entire study group, the mean value of carotid IMT, measured with omitting the visible plaques, was 0.66 (95%: 0.64–0.67) mm, with a range 0.4–1.3 mm. The median IMT value was 0.6 (Q1–Q3: 0.6–0.7) mm. The maximum intrapatient IMT difference was 0.6 mm and was observed in 1 study participant. Generally, the degree of carotid atherosclerosis was similar in both arteries, but in 51 (10.2%) patients a the side-to-side IMT difference >0.1 mm was noted. Patients in this specific subgroup were older [55.6 (51.9–59.3) vs. 49.2 (48.1–50.4) years; *p *< 0.001] and were more frequently men (72.6 vs. 55.0%; *p *< 0.05); however, there were no differences in BMI, pulse pressure, smoking status, the cause of CKD or the duration of the period of renal insufficiency. This “asymmetric” subgroup was characterized by a significantly greater presence of carotid plaques (72.6 vs. 53.2%; χ^2^ = 6.91, *p *< 0.01), including calcified lesions (62.8 vs. 44.5%; χ^2^ = 7.15, *p *< 0.05).

All study participants were divided by using the mean value of maximal carotid IMT, i.e., 0.66 mm. Table 2 shows the comparison of patients assigned to both groups. The structure of the primary cause of CKD was similar in both groups. IMT values were strongly associated with age (R = 0.498; *p *< 0.001). There were also positive correlations with BMI (R = 0.125; *p *< 0.01), pulse pressure (R = 0.220; *p *< 0.001) and the duration of renal insufficiency (R = 0.167; *p *< 0.001). Among the laboratory parameters, IMT was positively associated with blood hemoglobin level (R = 0.153; *p *< 0.001), OPG (R = 0.203; *p *< 0.001), IL-6 (R = 0.146; *p *< 0.01), serum sclerostin concentration (R = 0.198; *p *< 0.05) and negatively associated with serum Klotho concentration (R = –0.181; *p *< 0.05), but not with iPTH level.

**Table 2.** **The clinical characteristics of study subgroups based on the occurrence and type of atherosclerotic plaques visualized in both carotid arteries**.

Parameter	Study group according to the mean IMT value		*p*	Study groups according to the carotid plaque occurrence			*p*
	IMT max	IMT max		No plaques	Non-calcified	Calcified	
	<0.66 mm	≥0.66 mm		N = 224	N = 44	N = 232	
	N = 280	N = 220					
Age [years]	45.3 (43.9–46.6)	55.8 (54.3–57.3)	<0.001	42.7 (41.3–44.1)	49.3 (45.9–52.8)^∧∧∧^	57.0 (55.7–58.3) ###^∧∧∧^	<0.001
Gender [M/F]	138/142	146/74	<0.001	111/113	24/20	149/83##	<0.01
BMI [kg/$m^{2}$]	25.8 (25.2–26.4)	26.5 (25.9–27.1)	0.12	25.8 (25.1–26.5)	25.1 (23.8–26.4)	27.0 (26.4–27.6)${\#}^{\wedge }$	<0.05
Dialysis vintage [months]*	23 (14–42)	25 (14–41)	0.61	22 (13–40)	26 (16–38)	27 (15–45)	0.19**
Time after transplant [months]*	85 (52–115)	87 (70–119)	0.20	87 (57–116)	79 (25–104)	86 (70–120)	0.44**
Retransplant [n (%)]	21 (7.5)	21 (9.6)	0.40	19 (8.5)	3 (6.8)	20 (8.6)	0.92
Duration of renal insufficiency [years]*	10 (7–15)	12 (10–17)	<0.001	9.0 (7.0–12.0)	10.0 (7.5–15.5)	14.0 (10.0–18.0)	<0.001
Hypertension [n (%)]	240 (85.7)	200 (90.9)	0.08	190 (84.8)	37 (84.1)	218 (94.0)${\#\#}^{\wedge }$	<0.01
MAP [mmHg]	101 (100–103)	99 (98–101)	<0.05	100.0 (98–101)	100 (96–103)	101 (100–103)	0.35
Pulse pressure [mmHg]*	50 (45–60)	50 (40–59)	<0.001	50 (40–59)	50 (45–60)	50 (45–60)###	<0.05**
Number of antihypertensive drugs [n]*	2 (1–3)	2 (1–3)	0.06	2 (1–2)	2 (1–3)	2 (1–3)###	<0.001**
Diabetes [n (%)]	80 (28.5)	83 (37.8)	0.03	49 (21.9)	9 (20.4)	79 (34.1)##	<0.01
Coronary artery disease [n (%)]	21 (7.5)	49 (22.2)	<0.001	11 (4.9)	3 (6.8)	49 (21.1)${\#\#\#}^{\wedge }$	<0.001
Previous MACE [n (%)]	16 (5.7)	28 (12.7)	<0.01	10 (4.5)	1 (2.3)	43 (18.5)###^∧∧^	<0.001
Smoking status [%]	48 (17.1)	41 (18.6)	0.66	43 (19.2)	10 (22.7)	36 (15.5)	0.40
eGFR [mL/min/1.73 $m^{2}$]*	49.0 (36.1–67.7)	49.7 (37.9–68.8)	0.46	51.7 (38.4–68.3)	48.3 (38.5–63.0)	48.5 (34.8–66.9)	0.28**
Proteinuria ≥1 g/24 h [n (%)]	15 (5.4)	18 (8.2)	0.21	16 (7.1)	4 (9.1)	15 (6.5)	0.82
Glucose [mmol/L]*	5.0 (4.6–5.5)	5.2 (4.8–5.8)	<0.01	4.9 (4.6–5.5)	5.0 (4.4–5.4)	5.3 (4.8–5.9)#	<0.01**
Calcium [mmol/L]*	2.39 (2.30–2.50)	2.36 (2.27–2.45)	<0.05	2.4 (2.3–2.5)	2.4 (2.3–2.4)	2.4 (2.3–2.5)	0.25**
Phosphate [mmol/L]*	1.02 (0.87–1.18)	10.3 (0.87–1.15)	0.99	1.0 (0.9–1.2)	1.0 (0.8–1.2)	1.0 (0.9–1.1)	0.70**
iPTH [pg/mL]*	99 (68–171)	100 (60–148)	0.39	104 (70–174)	105 (70–149)	111 (66–177)	0.74**
IMT [mm]*	0.6 (0.5–0.6)	0.7 (0.7–0.8)	<0.001	0.6 (0.5–0.7)	0.7 (0.6–0.7)	0.7 (0.6–0.8)	<0.001**
Cholesterol [mmol/L]	5.3 (5.2–5.4)	5.4 (5.2–5.5)	0.33	5.3 (5.1–5.5)	5.4 (5.0–5.7)	5.3 (5.2–5.5)	0.94
Triglycerides [mmol/L]*	1.5 (1.0–2.0)	1.5 (1.0–2.2)	0.59	1.4 (1.0–2.0)	1.5 (1.1–2.0)	1.6 (1.1–2.2)	0.49**
Hyperlipidemia [n (%)]	145 (51.8)	117 (53.2)	0.76	116 (51.8)	22 (51.2)	127 (54.7)	0.75
Hyperuricemia [n (%)]	151 (52.9)	135 (61.4)	0.10	116 (51.8)	19 (43.2)	151 (65.1)##^∧∧^	<0.01
Hemoglobin [g%]	13.3 (13.0–13.5)	13.7 (13.5–14.0)	<0.01	13.4 (13.1–13.6)	13.6 (12.5–14.6)	13.6 (13.3–13.8)	0.53

Data presented as means and 95 % Confidence Intervals or frequencies, except *medians and Q1–Q3 values. Statistics: ANOVA or χ^2^ test, except **Kruskal-Wallis test. # *p *< 0.05 vs. no plaques; ## *p *< 0.01 vs. no plaques; ###* p <* 0.001 vs. no plaques; ^ *p *< 0.05 vs. non-calcified plaques; ^^ *p *< 0.01 vs. non-calcified plaques; ^^^ *p *< 0.001 vs. non-calcified plaques. BMI, body mass index; MAP, mean arterial pressure; MACE, major adverse cardio- and cerebrovascular event; eGFR, estimated glomerular filtration rate; iPTH, intact parathormon; IMT, intima-media thickness.

Univariate logistic regression analyses revealed that age, male sex, the presence of coronary artery disease or calcified plaques and hemoglobin level were associated with the presence of IMT ≥0.66 mm (Table 3).

**Table 3.** **Results of univariate logistic regression analyses for the IMT value and for the presence of calcified carotid plaques**.

	IMT			Calcified plaques		
	β	χ ^2^	*p*	β	χ ^2^	*p*
Age [years]	0.05	10.6	<0.01	0.12	162.4	<0.001
Male sex	1.63	9.4	<0.01	0.57	9.8	<0.01
BMI [kg/$m^{2}$]	0.04	0.63	0.43	0.06	8.6	<0.01
Hypertension	0.16	0.05	0.83	1.02	10.4	<0.01
Pulse pressure [mmHg]	0.01	0.5	0.48	0.03	16.2	<0.001
Number of antihypertensive drugs	0.03	0.03	0.85	0.42	30.9	<0.001
Coronary artery disease	1.46	4.67	<0.05	1.42	12.3	<0.001
Previous MACE	–0.29	0.09	0.78	1.72	17.7	<0.001
Calcium [mmol/L]	0.63	0.27	0.61	0.11	0.06	0.81
Glucose [mmol/L]	0.03	0.09	0.76	0.12	5.7	<0.05
Hyperuricemia	–0.30	0.38	0.54	0.62	10.4	<0.01
Hemoglobin [g%]	0.16	3.05	0.06	0.04	0.98	0.33
Duration of renal insufficiency period [years]	–0.04	0.81	0.39	0.10	36.9	<0.001
IMT [mm]	-	-	-	4.91	54.9	<0.001
Calcified plaques	1.71	12.3	<0.01	-	-	-

BMI, body mass index; MACE, major adverse cardio- and cerebrovascular event; IMT, carotid intima-media thickness.

Multivariate logistic regression analysis in the entire study group showed that age ($r_{\text{partial}}$ = 0.409; *p *< 0.001), male sex ($r_{\text{partial}}$ = 0.199; *p *< 0.001), and coronary artery disease ($r_{\text{partial}}$ = 0.139; *p *< 0.01) independently increased IMT ($R^{2}$ = 0.25).

### 3.3 Carotid Plaques

Among the 500 study patients, 276 (55.2%) had at least one plaque (non-calcified or calcified) in both carotids. Patients with the presence of plaques were significantly older [55.8 (54.5–57.0) vs. 42.7 (41.3–44.1) years; *p *< 0.001] in comparison with KTRs with no carotid atherosclerotic lesions. They more frequently were men (62.7 vs. 49.6%;* p <* 0.01) and were characterized by a greater BMI [26.5 (25.9–27.0) vs. 25.6 (24.9–26.3) kg/$m^{2}$, respectively; *p *< 0.01], IMT [Me: 0.70 (0.60–0.80) vs. Me: 0.60 (0.50–0.68) mm, respectively; *p *< 0.001], pulse pressure [Me: 50 (45–60) vs. Me: 50 (40–59) mmHg, respectively;* p <* 0.001] and duration of renal insufficiency [Me: 13 (10–18) vs. Me: 9 (7–12); *p *< 0.001].

**Fig. 1.**
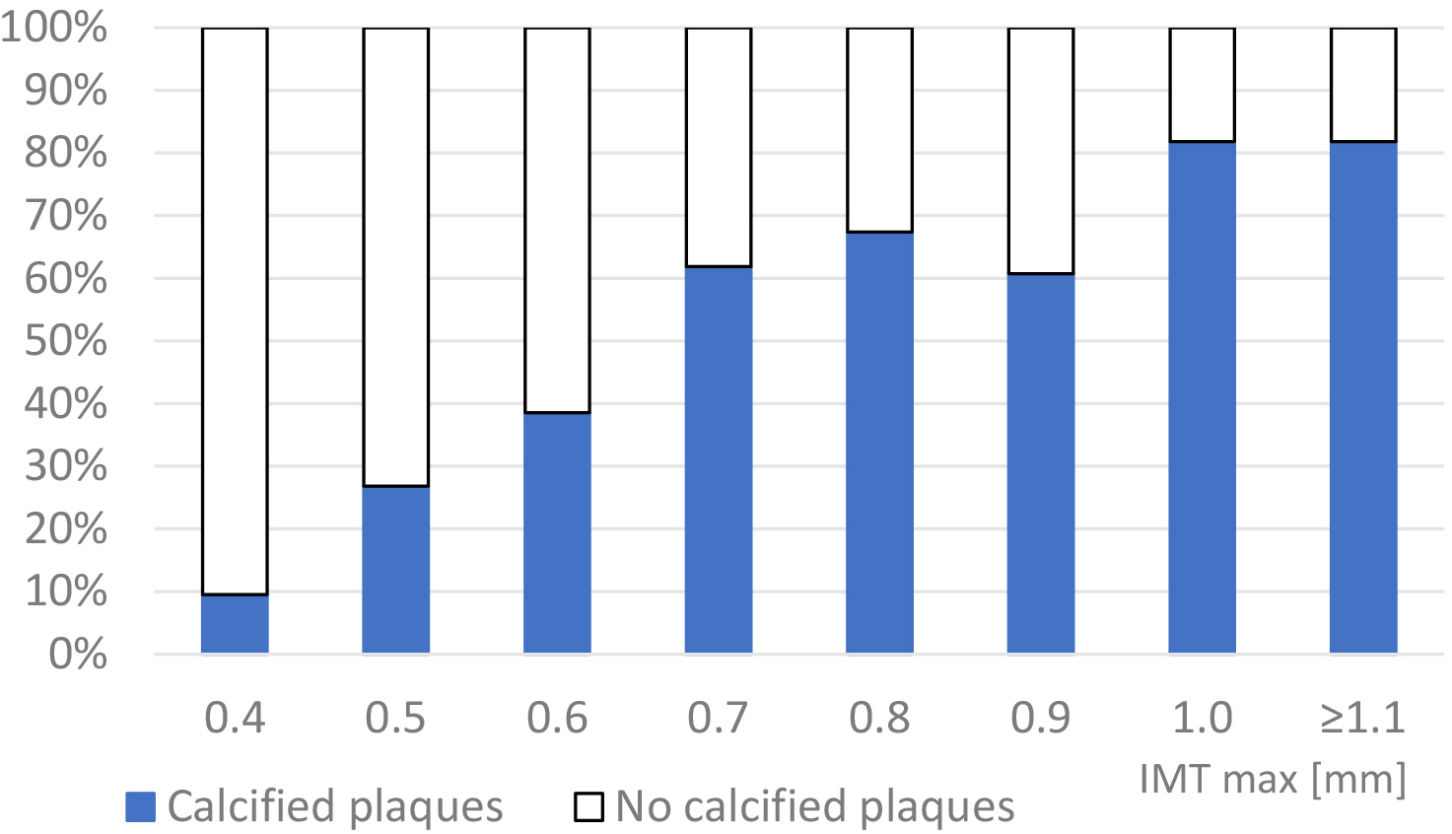
**The distribution of carotid calcified plaques in patients with different IMT max values**.

Calcified lesions were detected in 32.5% study subjects with an IMT ≤0.6 mm, including 17.9% of patients characterized by the presence of few calcified lesions and those with a heavily calcified carotid bulb. This finding was also present in 22.9% and 6.7% of subjects with an IMT ≤0.5 mm. Fig. 1 shows the distribution of calcified plaques in patients with different maximum IMT values.

All patients were divided into 3 groups, based on the presence and type of plaques detected in both carotid arteries. There were 224 (44.8%) patients without any plaque, 44 (8.8%) patients with only non-calcified plaque/plaques and 232 (46.4%) patients, in whom at least one calcified lesion was identified. The clinical characteristics of patients in these study groups are shown in Table 2. There were substantial differences between the study groups. However, except of age, where a significant increasing trend was noted across all 3 study groups (*p *< 0.001), other significant differences were noted between the calcified plaques group and one or both other groups (Table 2). Of note, the statistical strength of comparison between the non-calcified and calcified plaques groups was weakened by the low number of patients in the former group. There were no differences in the proportion of patients, in whom a parathyroidectomy was performed prior to the study (7 vs. 7 vs. 8%, respectively; *p* = 0.96).

In general, the occurrence of calcified plaques was associated with the primary cause of CKD (χ^2^: 12.2; *p *< 0.05). Patients with pyelonephritis had significantly less (χ^2^: 4.97; *p *< 0.05), whereas patients with hypertensive nephropathy significantly more (χ^2^: 8.3; *p *< 0.01) carotid calcified lesions as compared with other patients. Patients with pyelonephritis were significantly younger than all other groups (46.5 vs. 50.3 years; *p *< 0.05), but there was no age difference in case of patients with hypertensive nephropathy (52.5 vs. 50.0 years; *p* = 0.22).

Univariate logistic regression analyses revealed that age, male sex, BMI, IMT, pulse pressure, serum glucose level, the presence of hypertension, coronary artery disease, previous MACE, hyperuricemia, the number of antihypertensive drugs and the duration of renal insufficiency period prior to transplantation were associated with the presence of calcified carotid lesions (Table 3). There was also a significant relationship between the maximal carotid plaque score and the duration of the period of renal insufficiency (χ^2^: 52.7;* p <* 0.001).

**Fig. 2.**
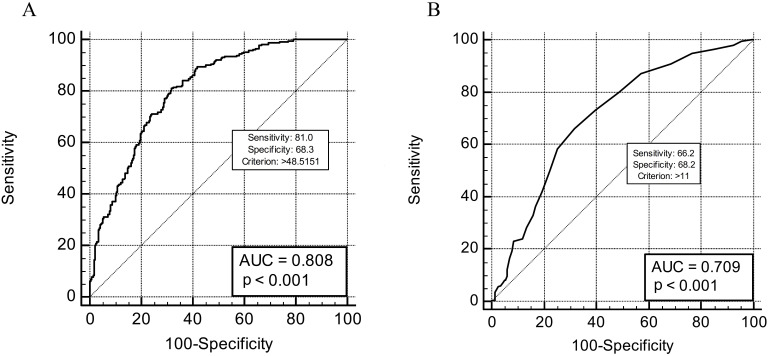
**The ROC analysis for recipient age (A) and duration of the period of renal insufficiency (B) which increased the risk for the occurrence of calcified lesions in the cohort of kidney transplant patients**.

Multivariate logistic regression analysis in the entire study group revealed that age ($r_{\text{partial}}$ = 0.544; *p *< 0.001), male sex ($r_{\text{partial}}$ = 0.127; *p *< 0.05), and the duration of the period of renal insufficiency ($r_{\text{partial}}$ = 0.235; *p *< 0.001) independently increased the risk for the presence of calcified lesions in carotid arteries ($R^{2}$ = 0.38). Notably, if we did not include the duration of the period of renal insufficiency among the potential independent variables, only age ($r_{\text{partial}}$ = 0.538; *p *< 0.001) and previous MACE ($r_{\text{partial}}$ = 0.130; *p *< 0.01) were confirmed in multivariate analysis ($R^{2}$ = 0.32). The ROC analysis revealed that age >48.5 years and the duration of the period of renal insufficiency >11 years increased the risk for the occurrence of calcified lesions with 81.0% and 66.2% sensitivity and 68.3% and 68.2% specificity, respectively (Fig. 2A,B).

In 319 study patients, the results of circulating markers of inflammation, vascular function and calcium-phosphate metabolism were also available. In this subgroup, there were significantly higher serum CRP levels in patients with calcified plaques [Me: 3.5 (1.5–7.3) vs. Me: 2.0 (1.2–5.2) mg/L; *p *< 0.01], whereas there were no differences in plasma OPG, ET-1, ADMA, ox-LDL, IL-6 and TNF-α levels (data not shown).

Plasma sclerostin and α-Klotho concentrations were measured in a subset of 146 study patients. Sclerostin levels were significantly higher [Me: 0.9 (0.7–1.1) vs. Me: 0.7 (0.6–0.1) ng/mL; *p *< 0.01] in patients with carotid calcified lesions, whereas the difference in α-Klotho levels [Me: 470 (392–577) vs. Me: 503 (440–616) pg/mL, respectively; *p* = 0.053] did not reach statistical significance.

Univariate logistic regression analyses revealed that serum CRP level (β = 0.05, χ^2^ = 5.9; *p *< 0.05), plasma OPG (β = 0.17, χ^2^ = 13.8; *p *< 0.001) and sclerostin (β = 1.82, χ^2^ = 12.8; *p *< 0.01) levels were associated with the presence of calcified carotid lesions. However, none of analyzed biomarkers independently influenced the occurrence of calcified plaques in the multivariate analysis.

### 3.4 Carotid Plaque Location

**Fig. 3.**
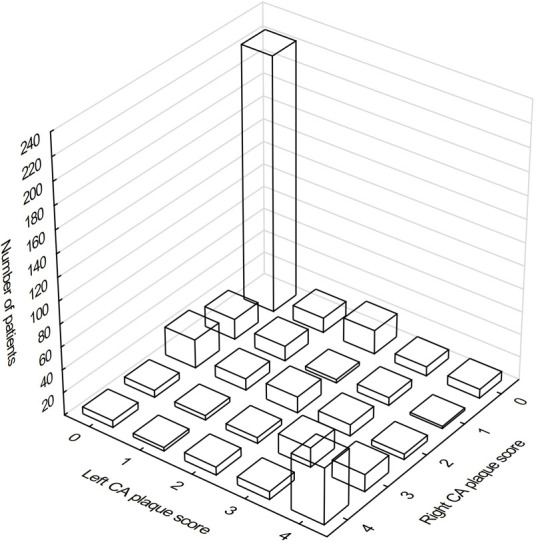
**The distribution of atherosclerotic lesions in carotid arteries according to the plaque score and side involved**. 0 denoted no plaques on the given side, 1—non-calcified lesions, 2—at least one calcified lesion, 3—few calcified lesions, 4—carotid bulb heavily covered by calcified lesions.

The atherosclerotic lesions distribution according to the plaque score and side involved is shown at Fig. 3.

Despite the comparable number of patients without any carotid lesion (274 vs. 279 at the right and left side, respectively), only 224 (44.8%) KTRs were free of plaques at both sides. In the group of 274 KTRs without plaques on the right side, 50 patients (18.2%) had atherosclerotic lesions on the left side, including 13 (4.7%) with uncalcified plaques and 37 (13.5%) with calcified plaques. Notably, 16 (5.8%) patients had an abundantly calcified left carotid bulb. In this group of 274 subjects, median left IMT was significantly greater in the subgroups with any ipsilateral plaques [Me: 0.6 (0.6–0.7); *p *< 0.01], calcified plaques [Me: 0.6 (0.6–0.7); *p *< 0.05] and abundantly calcified left carotid bulb [Me: 0.7 (0.6–0.8); *p *< 0.01] in comparison with subgroup without plaques [Me: 0.6 (0.5–0.6)].

Out of 279 patients without plaques on the left side, 55 patients (19.7%) had at least one atherosclerotic lesion on the right side, including 18 (6.5%) with uncalcified plaques and 37 (13.3%) with calcified plaques. Twelve patients (4.3%) had a heavily calcified right carotid bulb. Median right IMT was significantly greater in the subgroups with any ipsilateral plaques [Me: 0.6 (0.6–0.7); *p *< 0.001] and calcified plaques [Me: 0.6 (0.6–0.7); *p *< 0.001] in comparison with subgroup without plaques [Me: 0.6 (0.5–0.6)]. In contrast, there was no difference in IMT between subgroup with abundantly calcified right carotid bulb [Me: 0.6 (0.6–0.6)] as compared with the subgroup without plaques (*p* = 0.11).

## 4. Discussion

This study analyzed the characteristics and relationship of two different ultrasound-based measures of carotid atherosclerosis, IMT and carotid plaques, in stable KTRs. In contrast to several previous studies, the IMT measurements were performed manually (to avoid IMT overestimation), which enable us to investigate the two different atherosclerotic carotid entities separately and to define their independent risk factors without overlapping bias. Age and male sex were confirmed as common independent risk factors for the occurrence of both IMT and calcified carotid plaques. Additionally, the presence of coronary artery disease was associated with increased IMT, whereas the duration of the period of pre-transplant renal insufficiency increased the risk for carotid calcified plaques in multivariate analyses. Notably, none out of the numerous analyzed biomarkers was shown to be independently associated with IMT or plaque occurrence in the study cohort. This finding preclude the use of biomarkers as surrogates of carotid atherosclerosis in daily clinical practice. Finally, we also described in detail the asymmetric distribution of carotid lesions and the relationships between IMT and the presence of calcified carotid lesions in KTRs.

IMT is recognized as an useful tool for CV risk stratification and therapy monitoring [18]. In the Carotid Atherosclerosis Progression Study during a mean follow-up of 4.2 years, IMT was highly predictive for the incidence of stroke, myocardial infarction and death [19]. Moreover, for this combined end-point, hazard ratios were considerably higher in the younger (<50 years) than in the older age group. IMT was also shown to increase over a 6-year follow-up period especially in patients suffering from CV events [20]. In contrast, in the Rotterdam Study, adding IMT to a model for predicting the increased CV risk did not enhance its power, however the IMT ROC area (0.71) was comparable with other well-established risk factors, including previous MACE, diabetes, smoking, systolic blood pressure and cholesterol (ROC 0.65–0.72) [21]. In the present study, age, male gender and the presence of coronary artery disease were independently associated with greater IMT values, similarly to previous findings in the general [22] and hemodialysis [23] populations, despite one contrary report based on a small KTRs cohort [24].

Decreased renal function was shown to independently increase IMT (4), even in patients with no known kidney disease and normal and/or moderately decreased eGFR values [25, 26]. Of note, in the Suita Study cohort, multivariable-adjusted carotid IMT was significantly greater in CKD patients only in a hypertensive subgroup [27]. Nevertheless, despite the previously described modest IMT regression observed after kidney transplantation [28], the range of IMT in the present study was similar to the values reported in hemodialysis patients [29], as well as in other KTRs cohorts with comparable recipient age [30, 31]. Notably, in all study subjects, IMT was measured manually by a single investigator, within the optimal location at the far wall below the bulb, omitting any visible local thickening [32]. Otherwise, both the manual and computer-based, automated methods for IMT assessment often yield higher scores, as they may not omit local intimal thickening or plaques [22, 33, 34, 35]. This study protocol was chosen to avoid the above mentioned biases and allowed us to perform a more accurate analysis of interrelationship between IMT and carotid plaque burden. In the analyzed cohort, only a small subgroup showed substantial IMT left-right asymmetry, which is in line with earlier reports [36, 37]. Interestingly, those KTRs were older and were more frequently men compared to the rest of the analyzed cohort.

The assessment of carotid plaques is a distinct measure of atherosclerosis, with advancing age as the most predominant risk factor. With aging, a decrease in fibrous plaques and an increase in atheromatous plaques is observed [38]. This discrepancy may be explained by the differences in arterial remodeling in response to plaque accumulation among the different types of arteries [39]. Carotid bulb geometry was shown to be associated with plaque volume [40, 41]. Moreover, in arteries with plaques, wall shear stress was significantly lower than in the plaque-free vessel and was linked to endothelial dysfunction [42]. This may result in partly asymmetric distribution of carotid calcified lesions that was seen in some study patients, which was also reported in another large non-CKD study [43].

Thus, the assessment of carotid plaques, particularly calcified lesions, using ultrasound may provide additional stroke risk information beyond the measurement of luminal stenosis [44]. In a population-based study, calcified carotid plaques independently increased the risk of combined vascular outcomes, including ischemic stroke, even after adjustment for IMT [45]. The presence of CKD additionally increases the total and calcified plaque burden, both in dialysis and transplant patients [46]. Moreover, the negative association between eGFR and the prevalence of carotid plaques was observed even in patients without CKD [25]. This effect is mainly mediated by phosphate retention, which constitutes an early trigger for the development of secondary hyperparathyroidism and accelerated macrovascular disease [47]. Such a mechanism is further confirmed by a consistent relationship between serum phosphate (even in the normal range) and CVD [48, 49], as well as with multiregional vascular calcification [50], and by the independent relationship between the duration of renal insufficiency (with concomitant impairment of urinary phosphate elimination) and calcified plaques found in the present study. This reciprocal relationship of vascular calcifications within different locations in CKD patients was also confirmed in other studies [22, 29, 51, 52].

In kidney transplant patients, the occurrence of carotid lesions was previously showed to be associated with age and the occurrence of arterial hypertension [53]. Importantly, despite satisfactory transplant organ function, substantial progression of carotid plaques was reported, which was associated with age, smoking, dialysis vintage and hyperphosphatemia [54]. In the present study we found that, except for age and male sex, only the duration of pre-transplant renal insufficiency independently increased the burden of calcified carotid plaques, and as we excluded the latter variable, only age and previous MACE remained significant. This is in line with another report, where the severity of the carotid plaque score was significantly higher in the MACE group than in the MACE-free group in asymptomatic CKD patients [55]. Similarly, age and coronary artery calcification score were independently associated with carotid plaques in dialysis patients [29]. Interestingly, in some reports a significantly greater plaque prevalence in dialysis patients in comparison with healthy controls was identified despite similar IMT [45, 56]. It is also worth noticing that in our cohort, there was a substantial percentage of patients with IMT ≤0.6 mm, in whom we found a high burden of calcified carotid plaques. More importantly, the accuracy for detecting carotid plaques was higher than for detecting abnormal IMT in examinations performed in a routine outpatient setting in a non-CKD cohort [57]. In the large Angina Prognosis Study in Stockholm, carotid IMT was a weak predictor for events, whereas carotid plaques were related to CV death or non-fatal myocardial infarction [58]. In a recent metanalysis, plaque assessment was found to be a better CV risk predictor than IMT in a non-CKD population and one large observational study provided evidence for its similar potential in CKD patients [59]. These studies suggest the greater utility of carotid plaque assessment compared to carotid IMT measurement, as a prognostic indicator for CV risk in CKD and KTRs.

Disappointingly, when we analyzed the potential associations of numerous biochemical or hormonal markers with IMT or the occurrence of carotid plaques, none of investigated inflammatory, vascular or calcium-phosphate parameters was confirmed as an independent variable in multivariate analyses. Previously, atherosclerotic plaque occurrence and progression were found to be associated with higher IL-6 levels [60] and α-Klotho polymorphism [61] in CKD patients. Plaque burden was also related to ET-1, OPG and nitric oxide metabolite levels in small cohorts of dialysis patients [62, 63, 64] and with CRP level in KTRs [65]. However, a majority of biomarkers included in the present analysis have not been previously examined in kidney transplantation patients.

The main limitation of our analysis is its cross-sectional character and inclusion of 3 KTRs cohorts. However, there was a high uniformity in the methodology of these 3 cohorts, with all ultrasound examinations performed by a single investigator, using the same measurement protocol. In our study cohort, several clinical characteristics and several biochemical parameters were not available for each of study patients. Only the smoking status at the time of the study was available, whereas we have no data concerning the lifetime smoking habits. This may have explained why smoking was not found to be an independent parameter which influenced IMT or the occurrence of calcified plaques in the univariate or multivariate analyses. Finally, as calcium-phosphate metabolism before and during dialysis therapy is the crucial factor for accelerated calcification of the vessel wall, it would have been important to analyze data concerning the maximum pre-transplant phosphate and iPTH levels, as well as the use and effect of phosphate-lowering regimens, which were not available in our study group.

## 5. Conclusions

In this study, we described in detail the distribution of two different atherosclerotic measures—IMT and carotid plaques—in a large cohort of stable kidney transplant recipients. In addition, the profiles of different clinical risk factors associated with both those vascular entities were identified. We large side-to-side differences in IMT values and carotid plaque distribution in a substantial percentage of KTRs, which presents a high epidemiologic burden for carotid and general atherosclerosis. None of the analyzed vascular and calcium-phosphate metabolism biomarkers was associated with any of the carotid atherosclerosis measurements. Due to the high risk for CV complications and death among recipients of successful kidney transplants, the independent assessment of both IMT and calcified carotid lesions should be advocated, as it may increase the ability to identify those KTRs with the highest CV risk.
